# Omalizumab as add‐on therapy in a patient with severe asthma and OSA

**DOI:** 10.1002/rcr2.518

**Published:** 2020-02-07

**Authors:** Giulia Scioscia, Enrico Buonamico, Maria Pia Foschino Barbaro, Donato Lacedonia, Roberto Sabato, Giovanna Elisiana Carpagnano

**Affiliations:** ^1^ Institute of Respiratory Diseases, Department of Medical and Surgical Sciences University of Foggia Foggia Italy; ^2^ Institute of Respiratory Diseases, Department of Neurosciences University of Bari Bari Italy; ^3^ Respiratory Diseases Section, Department of Basic Medical Sciences Neuroscience and Sense Organs; University of Bari Bari Italy

**Keywords:** Biological therapy, lung function, obstructive sleep apnoea, polysomnography, severe asthma

## Abstract

Obstructive sleep apnoea (OSA) syndrome, the most frequent sleep‐disordered breathing, is a comorbidity of asthma, whose prevalence covers about 49.5% of asthmatic adult patients. A 61‐year‐old female patient, affected by severe allergic asthma and obesity, started treatment with omalizumab and underwent polysomnography showing a severe OSA pattern (apnoea/hypopnoea index (AHI): 72.7). After six months, she showed functional improvement and good asthma symptoms control and underwent a new polygraphy for the persistence of the night symptoms which showed an ameliorated, despite still severe, OSA pattern (AHI: 31.9). The patient obtained complete polygraphic normalization after adequate positive airway pressure (PAP) titration. While bronchodilator efficacy in chronic obstructive pulmonary disease (COPD)/OSA overlap syndrome has been proven in raising nocturnal oxygen saturation, there is no such evidence about biological therapy in patients affected by severe asthma and OSA. This is the first documented case report that demonstrates a possible role of omalizumab in improving the OSA pattern in a patient affected by severe asthma and OSA.

## Abstract 2

We report the case of a 61‐year‐old severe allergic asthmatic woman (body mass index (BMI): 38) who was followed up by our outpatient clinic for one year. She started treatment with omalizumab and underwent polysomnography showing a severe obstructive sleep apnoea (OSA) pattern (apnoea/hypopnoea index (AHI): 72.7). After six months, she showed functional improvement and good asthma symptoms control and underwent a new polygraphy for the persistence of the night symptoms which showed an ameliorated, despite still severe, OSA pattern (AHI: 31.9). The patient obtained complete polygraphic normalization after adequate positive airway pressure (PAP) titration. This is the first documented case report that demonstrates a possible role of omalizumab in improving the OSA pattern in a patient affected by severe asthma and OSA.

## Introduction

Asthma is a well‐known chronic respiratory disease characterized by chronic airways inflammation. The goal in the management of the asthmatic patient is to reach disease control, assessed by questionnaires (Asthma Control Test (ACT) or Asthma Control Questionnaire (ACQ)) and recent anamnesis of re‐exacerbations 1.

During the last decade, a new approach has emerged in severe allergic asthma: biological therapy with humanized anti‐immunoglobulin (Ig) E antibody omalizumab, followed by other biological molecules that target mediators involved in eosinophilic asthma, helping to reach disease control, to lower the annual bronchial re‐exacerbation number also with a corticosteroid‐sparing effect.

Obstructive sleep apnoea (OSA) is the most frequent among sleep‐disordered breathing. Sleep apnoea syndrome is defined by several apnoeic/hypopnoeic events and is associated with not only cardiovascular morbidity and mortality, but also excessive daytime sleepiness which can lead to automobile accidents.

OSA is a well‐known comorbidity of asthma 1. An important American multicentre real‐life study found prevalence of OSA in omalizumab‐eligible patients being 14.6% 2. A 2017 large meta‐analysis reported prevalence of OSA in adult asthma patients as 49.5% and the odds of having OSA by asthma patients was 2.64 times higher than in controls 3. Prevalence of OSA seems correlated to asthma burden, following the evidence that OSA prevails in patients with severe asthma than in those with moderate asthma 4.

## Case Report

We report the case of a 61‐year‐old severe allergic asthmatic woman (body mass index (BMI): 38) who was followed up by our outpatient clinic for one year. At the first visit, the patient showed an uncontrolled asthma (ACT: 6), reporting frequent cough, wheezing, and night‐time awakening due to asthma symptoms.

The patient referred history of allergic oculo‐rhinitis during the spring season, probably due to her sensitization to olive tree pollen. During this season, the patient takes antihistamine drugs and nasal corticosteroid sprays as needed.

Spirometry was performed showing mild obstruction (forced expiratory volume in 1 sec (FEV_1_): 1.38 mL—72%; forced vital capacity (FVC): 2.06 mL—90%; Tiffeneau index: 0.67), with a negative bronchodilation test (FEV_1_: +10% and +180 mL). Eosinophils count on peripheral blood was 540 cells/μL and total IgE count paper radio immuno sorbent (PRIST) was 39 kU/L. The patient reported high oral corticosteroids usage (4–5 months per year). Inhaled therapy at admission was adjusted following Global Initiative for Asthma (GINA) guidelines with the introduction of tiotropium 5 μg/day with Spiriva Respimat, Boehringer Ingelheim Pharmaceuticals, Inc. device, mometasone 400 mcg/twice a day, and montelukast 10 mg tablet daily but long‐acting beta‐2 agonists (LABAs) were avoided because the patient referred tremors and palpitations after salbutamol and formoterol inhalation. At the first month follow‐up visit, the clinical situation was similar despite the attempts to optimize control therapy, and still symptoms were far from being controlled (ACT: 14, ACQ: 4.16). At this point, based on a discussion about the risks and benefits of every possible solution, a step up in anti‐asthmatic therapy with the humanized anti‐IgE antibody omalizumab at a dose of 150 mg every four weeks (according to weight and total IgE) was proposed to the patient and she accepted.

During the diagnostic/therapeutic process, a clinical picture of OSA emerged, with reported night‐time snoring and apnoeas, daytime fatigue, memory impairment, and mood disorders (Epworth Sleepiness Scale (ESS): 12; snoring, tiredness, observed apnea, high pressure (STOP Bang): 6). For this reason, the patient had sleep polygraphy showing a severe OSA clinical picture (apnoea/hypopnoea index (AHI): 72.7, oxygen desaturation index (ODI): 68, percent sleep time spent with SpO2 <90% (TST90): 19.7%, mean peripheral capillary oxygen saturation (SpO_2_): 91%, total sleep time: 437.5 min (7 h and 20 min), AHI in supine position: 89.9 (59% sleep time), AHI in left decubitus: 50.9 (37% sleep time), and AHI in right decubitus: 26.1 (4% sleep time)). Continuous positive airway pressure (CPAP) therapy was proposed to the patient but she did not accept as she had the purpose to lose weight through a dietetics scheme.

At the first control visit after four months from the first omalizumab administration, the patient showed a controller asthma (ACT: 21, ACQ: 0.33) and remarkable improvement in spirometric values (FEV_1_ 2.23 mL—117%; FVC: 2.85 mL—125%; Tiffeneau index: 0.78).

At the second control visit, after six months from the first omalizumab administration, the patient still showed a controlled asthma (ACT: 21) with a corroborating lung function (FEV_1_: 1.94 mL—103%; FVC: 2.52 mL—112%; Tiffeneau index: 0.77), but she referred the persistence of night‐time snoring and apnoeas. Therefore, a new basal outpatient polygraphy was performed, still showing a severe OSA pattern, but deeply improved since the last one performed, with AHI 31.9 (−56.12%), ODI 29 (−57.35%), mean arterial oxygen saturation (SaO_2_) 95% (+4%), TST90 12% (−7.7%), total sleep time 325.1 min (5 h and 25 min), AHI in supine position 41 (44% sleep time), AHI in left decubitus 18.8 (24% sleep time), and AHI in right decubitus 29.3 (33% sleep time); so she accepted CPAP. It is remarkable to point out that the patient did not undergo any weight loss from the last polysomnography, nor upper respiratory tract surgery. To explore daytime symptoms related to OSA, a new ESS was submitted to the patient and the result was 9, still high but sensitively reduced compared to the previous result.

After PAP titration to 8.5 cmH_2_O, the sleep polygraphy showed a complete normalization of the OSA syndrome (AHI: 3.0, ODI: 2.8, mean SaO_2_: 93%, TST90: 0.4%).

## Discussion

While bronchodilator therapy in chronic obstructive pulmonary disease COPD–OSA overlap patients has long proved its efficacy in the improvement of nocturnal oxygen saturation, discordant results have emerged about the role of asthma control therapy in OSA management, with evidence of linear dose‐dependent relationship of inhaled corticosteroids (ICS) usage with habitual snoring and high OSA risk 5.

In conclusion, we found, in this particular case, that biological control therapy for severe asthma with omalizumab decisively helped in the improvement of nocturnal obstructive clinical picture. After reaching asthma control and normal spirometric values, in the absence of other confounding changes, the patient's baseline polygraphy performed after the introduction of omalizumab showed a halved AHI compared to the previous examination performed through an uncontrolled asthma picture, despite still showing a severe OSA.

Although night‐time variability between the two polygraphies may explain the findings, it is possible that biological therapy for asthma, through an ameliorated disease control, may have improved OSA‐related parameters. Our case supports the consideration of further dedicated research into this area.

### Disclosure Statement

Appropriate written informed consent was obtained for publication of this case report and accompanying images.
